# Acupuncture for migraine prophylaxis

**DOI:** 10.1590/1516-3180.20151336T1

**Published:** 2015-04-14

**Authors:** Klaus Linde, Gianni Allais, Benno Brinkhaus, Eric Manheimer, Andrew Vickers, Adrian R. White

## Abstract

**BACKGROUND:**

Acupuncture is often used for migraine prophylaxis but its effectiveness is still controversial. This review (along with a companion review on 'Acupuncture for tension-type headache') represents an updated version of a Cochrane review originally published in Issue 1, 2001, of The Cochrane Library.

**OBJECTIVES:**

To investigate whether acupuncture is a) more effective than no prophylactic treatment/routine care only; b) more effective than 'sham' (placebo) acupuncture; and c) as effective as other interventions in reducing headache frequency in patients with migraine.

**METHODS:**

*Search methods:* The Cochrane Pain, Palliative & Supportive Care Trials Register, CENTRAL, MEDLINE, EMBASE and the Cochrane Complementary Medicine Field Trials Register were searched to January 2008.

*Selection criteria:* We included randomized trials with a post-randomization observation period of at least 8 weeks that compared the clinical effects of an acupuncture intervention with a control (no prophylactic treatment or routine care only), a sham acupuncture intervention or another intervention in patients with migraine.

*Data collection and analysis: *Two reviewers checked eligibility; extracted information on patients, interventions, methods and results; and assessed risk of bias and quality of the acupuncture intervention. Outcomes extracted included response (outcome of primary interest), migraine attacks, migraine days, headache days and analgesic use. Pooled effect size estimates were calculated using a random-effects model.

**MAIN RESULTS:**

Twenty-two trials with 4419 participants (mean 201, median 42, range 27 to 1715) met the inclusion criteria. Six trials (including two large trials with 401 and 1715 patients) compared acupuncture to no prophylactic treatment or routine care only. After 3 to 4 months patients receiving acupuncture had higher response rates and fewer headaches. The only study with long-term follow up saw no evidence that effects dissipated up to 9 months after cessation of treatment. Fourteen trials compared a 'true' acupuncture intervention with a variety of sham interventions. Pooled analyses did not show a statistically significant superiority for true acupuncture for any outcome in any of the time windows, but the results of single trials varied considerably. Four trials compared acupuncture to proven prophylactic drug treatment. Overall in these trials acupuncture was associated with slightly better outcomes and fewer adverse effects than prophylactic drug treatment. Two small low-quality trials comparing acupuncture with relaxation (alone or in combination with massage) could not be interpreted reliably.

Authors' conclusions: In the previous version of this review, evidence in support of acupuncture for migraine prophylaxis was considered promising but insufficient. Now, with 12 additional trials, there is consistent evidence that acupuncture provides additional benefit to treatment of acute migraine attacks only or to routine care. There is no evidence for an effect of 'true' acupuncture over sham interventions, though this is difficult to interpret, as exact point location could be of limited importance. Available studies suggest that acupuncture is at least as effective as, or possibly more effective than, prophylactic drug treatment, and has fewer adverse effects. Acupuncture should be considered a treatment option for patients willing to undergo this treatment.

**This abstract is available free of charge from:** http://onlinelibrary.wiley.com/doi/10.1002/14651858.CD001218.pub2/abstract
